# Earing Prediction of AA5754-H111 (Al-Alloy) with Linear Transformation-Based Anisotropic Drucker Yield Function under Non-Associated Flow Rule (Non-AFR)

**DOI:** 10.3390/ma17153865

**Published:** 2024-08-05

**Authors:** Xiang Gao, Zhen Zhang, Zhongming Xu, Xinming Wan, Songchen Wang, Naveed Muhammad Mubashir

**Affiliations:** 1 College of Mechanical and Vehicle Engineering, Chongqing University, Chongqing 400044, China; 2 China Automotive Engineering Research Institute Co., Ltd., Chongqing 401122, China; wanxinming@caeri.com.cn; 3 School of Mechanical Engineering, Xi’an Jiaotong University, Xi’an 710049, China

**Keywords:** numerical simulation, non-associated flow rule, plastic deformation, cylinder drawing deep, anisotropy

## Abstract

The yield behavior of aluminum alloy 5754-H111 under different stress conditions for three kinds of plastic work is studied using an anisotropic Drucker model. It is found that when the plastic work is 30 MPa, the anisotropic Drucker model has the most accurate prediction. Comparing the Hill48 and Yld91 models with the Drucker model, the results show that both the anisotropic Drucker and Yld91 models can accurately predict the yield behavior of the alloy. Cylinder drawing finite element analysis is performed under the AFR, but it is not possible to accurately predict the position and height of earing appearance. The anisotropic Drucker model is used to predict the earing behavior under the non-AFR, which can accurately predict the earing phenomenon. Numerical simulation is conducted using three different combinations of yield functions: the anisotropic yield function and the anisotropic plastic potential function (AYAPP), the anisotropic yield function and the isotropic plastic potential function (AYIPP), and the isotropic yield function and the anisotropic plastic potential function (IYAPP). It is concluded that the influence of the plastic potential function on predicting earing behavior is more critical than that of the yield function.

## 1. Introduction

Due to the continuous development of metallic materials, the application of isotropic Mises and the Tresca yield criterion to describe yield by shear stress-induced yield has been significantly limited. Consequently, in recent decades, various yield criteria have been proposed to characterize the anisotropic behavior during the deformation process of plates. Hill proposed the Hill48 yield criterion [1] in 1948 on the basis of the Mises yield criterion to describe the anisotropy of the yield stress of metallic materials. Esmaeilizadeh et al. [2] applied the von Mises model and the Hill-1948 model to predict the force–displacement curve of AA1200, demonstrating the applicability of the anisotropic yield criterion. On the basis of the Hill48 yield criterion, many anisotropic yield criteria for linear transformations of stress tensors in different ways have been proposed in recent years, such as Yld89 [3], Yld91 [4], Yld2000-2D [5], Yld2004-18P [6], BBC2000 [7], CPB2006 [8], pYld2000-2d [9], rYld2004 [10], etc. These yield criteria incorporate additional anisotropic parameters into the linear transformation of the stress tensor to more accurately describe the anisotropic behavior of metal sheets. In addition to the above yield criteria based on the linear transformation type of the stress tensor, many yield criteria based on stress invariants and stress offset tensors were also proposed. Drucker first proposed the construction of yield equations in the form of stress invariants in 1949 [11]. Cazacu and Balart extended the Drucker yield criterion to orthotropy by writing the stress skew tensor as an orthotropic form in 2001 [12]. In 2004 [13], Cazacu and Balart proposed a yield criterion to describe asymmetry for pressure-insensitive materials. Yoon et al. [14] proposed a yield function in the form of three stress invariants. Lou et al. proposed a series of yield functions that can calibrate BCC and FCC materials [15], simulate strength difference effects [16,17], and characterize anisotropy in tensile compression [18]. Recently, anisotropic hardening functions [19,20,21,22,23,24,25,26,27,28,29,30] were also proposed to describe yield surface evolution with respect to plastic strain.

The plastic flow law is divided into the associated flow rule (AFR) and the non-associated flow rule (non-AFR). In the AFR, it is assumed that the plastic potential function of the material is the same as the yield function, while in the non-AFR, it is assumed that the plastic potential function and the yield function of the material are independent of each other. Bridgman [31] concluded through experiments that hydrostatic pressure has no effect on the yield behavior of metal materials, and the volume of materials remains unchanged during plastic deformation. Based on this, the researchers believe that metal materials comply with the AFR. Li and Richmond [32] found that plastic deformation is inherently unstable in initial homogeneous materials that follow the non-AFR. Brünig and Obrecht [33] modified Schmid’s law and found the AFR to be irrational. In the AFR, it is difficult to use a function to describe both the anisotropic plastic flow and the anisotropic yield behavior of the material. Therefore, most researchers focus on the non-AFR.

Stoughton and Yoon [34] proposed to consider the hydrostatic pressure-sensitive non-AFR model to explain the asymmetric effect of tensile compression of materials. Park and Chung [35] established a non-AFR model based on the Yld2000-2d yield function and predicted 6 or 8 ears in cylinder drawing forming. Huang and Lu [36] found that the punch load increased with the increase in the punch stroke, and after the load reached the maximum, the billet continued to deform with the increase in the punch stroke. In a deep drawing simulation, Gao et al. [37] found that the hardening index and yield stress had a more significant effect on thickness change and equivalence change. Taherizadeh et al. [38] proposed a mixed-hardening non-AFR model, which made more accurate predictions of the shape of the deep earing and the amount of barrel wall springback. Liu et al. [39] applied electromagnetic forming technology to cylinder deep drawing and found that electromagnetic-assisted deep drawing can significantly improve the formability of aluminum cylindrical parts. Zhang et al. [40] used Yld89 to find that the thickness of the gap generator blank had the greatest effect on the thinning of the bottom blank. The effects of fracture mode, ultimate tensile ratio, thickness distribution [41], and coefficient of friction [42] in deep drawing tests have also been studied in depth. Zhang et al. [43] predicted the earing shape of AA6016 T4 by different yield functions under the non-AFR. In summary, the non-AFR has a wider range of applications, and the research on the deep drawing forming process has been very deep. However, the degree of influence of the yield function and the plastic potential function in predicting earing behavior under the non-AFR is not studied well.

In this paper, the Hill48 and Yld91 models based on stress invariants and the Drucker model based on stress skew tensors are used to predict the yield behavior of AA5754-H111. Through cylinder drawing test and simulation, the accuracy of the above yield model is verified, and the accuracy and time of the above three models are evaluated. To explore the influence of yield function and plastic potential function on the earing behavior of the alloy under the non-AFR, the research focuses on the Drucker model. The effects of yield function and plastic potential function on the earing behavior of the alloy under the non-AFR are discussed based on the Drucker model.

## 2. Experiment

**Figure 1:**
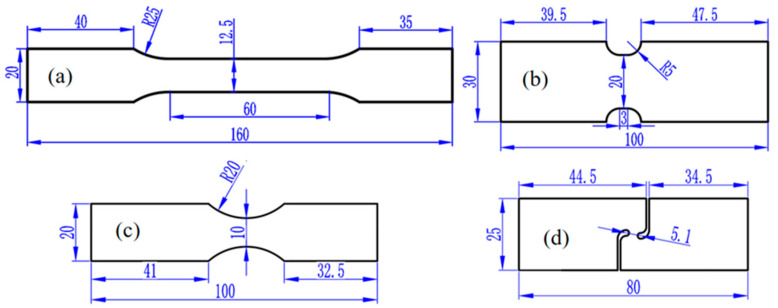
Four kinds of specimens for different stress states: (**a**) dogbone specimens; (**b**) notched specimens with R = 5 mm; (**c**) notched specimens with R = 20 mm; (**d**) in-plane shear specimens. The dimensions are in mm.

This section describes the acquisition of the test data of AA5754-H111 from different angles under various stress states, facilitating the study of the aluminum alloy’s mechanical properties under varying conditions. The alloy was a wrought product straightened after annealing to meet straightness tolerances with a strain lower than the amount required for a controlled H11 temper. Four types of specimens, namely (a) dogbone specimens, (b) notched specimens with R = 5 mm, (c) notched specimens with R = 20 mm, and (d) in-plane shear specimens, were cut from aluminum plates with a thickness of 3 mm using laser processing. These are shown in Figure 1. Due to the texture formed in plastic manufacturing processes, the mechanical properties are different for different loading directions, which is referred to as the anisotropy of metals. These four specimen types were cut from aluminum plates at three different angles of 0°, 45°, and 90° from the rolling direction to explore the anisotropy of AA5754-H111 under different stress states. In Figure 1a,c, the gauge distance between the dogbone specimens and the notched specimens with R = 20 mm is 30 mm, while in Figure 1b,d the gauge distance between the notched specimens with R = 5 mm and the shear specimens is 20 mm. Before the test, irregular sized speckles were evenly sprayed on all specimens to facilitate the DIC system in capturing the test images. The load on the tensile specimen was measured by force transducers and transmitted to the XTOP DIC system. The tensile speeds of the various types of specimens are shown in Table 1. To ensure the accuracy of the test results, each specimen was prepared with multiple identical specimens for repeated testing.

**Figure 2:**
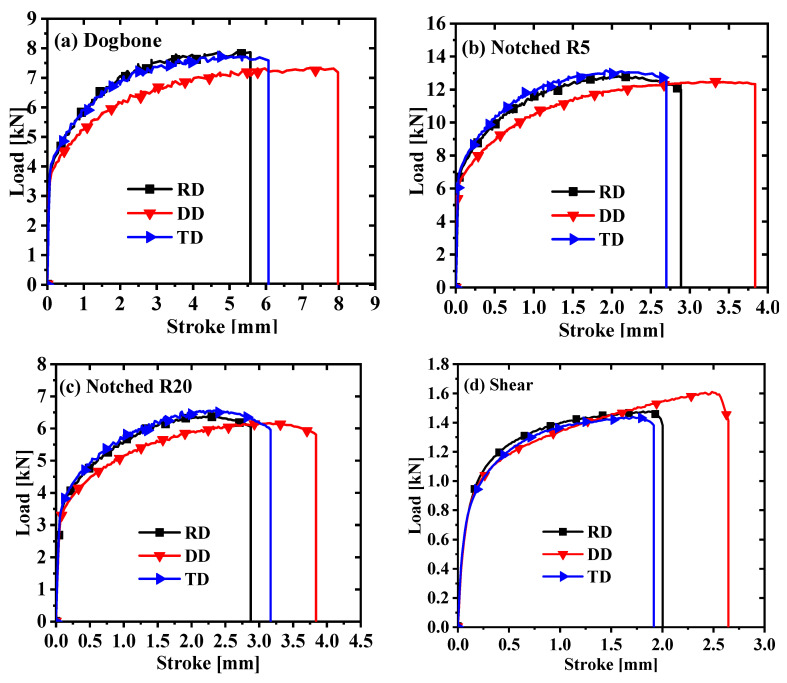
Force–displacement curves for four stress states: (**a**) dogbone specimens; (**b**) notched specimens with R = 5 mm; (**c**) notched specimens with R = 20 mm; (**d**) in-plane shear specimens.

**Figure 3:**
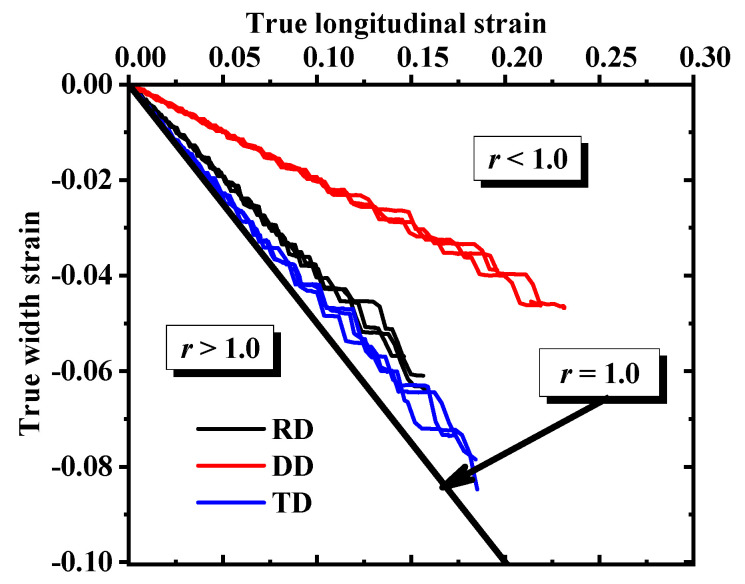
Dogbone test length direction strain and width direction strain.

Repeatability tests were conducted on four samples and the best set of curves was selected, as shown in Figure 2. Figure 2 reveals that AA5754-H111 displays clear anisotropy in the four stress states; notably, the curve in the DD direction is the most different compared with the other directions. The DIC system could detect and calculate the transverse and longitudinal strains in the three directions of the dogbone test, as shown in Figure 3. The measured transverse strain and longitudinal strain were used to calculate the R-values in the three directions, and the R-value calculation formula is as follows:

(1) $$R=\frac{\varepsilon_{w}}{\varepsilon_{t}}$$

In plastic deformation, the volume unchanged condition obtains the following:

(2) $$\varepsilon_{w}+\varepsilon_{t}+\varepsilon_{l}=0$$

In Equation (2), the strain along the width, thickness, and length of the dogbone test, substituting Equation (2) into Equation (1) yields the following:

(3) $$R=-\frac{\varepsilon_{w}}{\varepsilon_{w}+\varepsilon_{l}}=-\frac{s}{s+1}$$

where $s=\frac{\varepsilon_{w}}{\varepsilon_{t}}$. The R-values obtained by Equation (3) are summarized in Table 2.

## 3. Results

### 3.1. Hill48 Yield Criterion

The Hill48 quadratic yield criterion [1] is widely utilized to describe the plane anisotropy of sheets due to its simple calculation, especially in sheet metals where the forming process often involves a plane stress state. The Hill48 yield equation is expressed in the plane stress state as follows:

(4) $$2f\left(\sigma_{\mathrm{ij}}\right)=\left(G+H\right)\sigma_{x}^{2}-2H\sigma_{x}\sigma_{y}+\left(F+H\right){\sigma_{y}}^{2}+2N\tau_{\mathrm{xy}}^{2}=1$$

where F, G, H, and N are anisotropic constants related to the yield properties of the material; σ_x_ and σ_y_ are normal stresses, and σ_xy_ is the shear stress in the xy plane. The stress components in the Cartesian coordinate system (anisotropy axes x, y, z, with x-axis) are formulated as follows:

(5) $$\sigma_{x}=\sigma_{\theta }\cos^{2}\theta ,\;\sigma_{y}=\sigma_{\theta }\sin^{2}\theta ,\;\tau_{\mathrm{xy}}=\sigma_{\theta }\sin\theta \cos\theta$$

where σ_θ_ is the uniaxial tensile stress along θ, and θ is the angle between the direction of the stress spindle and the direction of rolling. The uniaxial tensile yield stress σ_θ_ is computed by the Hill48 yield function as below:

(6) $$f\left(\sigma_{\mathrm{ij}}\right)=\left(\left(G+H\right)\cos^{4}\theta -2H\sin^{2}\theta \cos^{2}\theta +\left(F+H\right)\sin^{4}\theta +2N\sin^{2}\theta \cos^{2}\theta \right)\sigma_{\theta }^{2}=1$$

The R-values of the three angles are expressed as follows [1]:

(7) $$R_{0}=\frac{H+H\tan^{2}\theta \sin^{2}\theta +N\sin^{2}\theta -\left(F+G+3H\right)\sin^{2}\theta }{G+F\tan^{2}\theta }=\frac{H}{G}$$

(8) $$R_{45}=\frac{H+H\tan^{2}\theta \sin^{2}\theta +N\sin^{2}\theta -\left(F+G+3H\right)\sin^{2}\theta }{G+F\tan^{2}\theta }=\frac{2N-\left(F+G\right)}{2\left(F+G\right)}$$

(9) $$R_{90}=\frac{H+H\tan^{2}\theta \sin^{2}\theta +N\sin^{2}\theta -\left(F+G+3H\right)\sin^{2}\theta }{G+F\tan^{2}\theta }=\frac{H}{F}$$

### 3.2. Drucker Yield Criterion

Drucker first proposed in 1949 [11] to consider the effect of the third stress invariant coupled to the von Mises function:

(10) $$f\left(\sigma_{\mathrm{ij}}\right)=a{\left(J_{2}^{3}-cJ_{3}^{2}\right)}^{\frac{1}{6}}={\bar{\sigma }}_{D}$$

The deviatoric stress tensors J_2_ and J_3_ are expressed as follows:

(11) $$J_{2}=\frac{1}{2}s_{\mathrm{ij}}s_{\mathrm{ij}}=\frac{1}{6}\left[{\left(s_{1}-s_{2}\right)}^{2}+{\left(s_{2}-s_{3}\right)}^{2}+{\left(s_{3}-s_{1}\right)}^{2}\right]$$

(12) $$J_{3}=\det\left(s_{\mathrm{ij}}\right)=s_{1}s_{2}s_{3}$$

In the above equations, J_2_ is the second invariant of the deviatoric stress tensor; J_3_ is the third invariant of the deviatoric stress tensor; a is determined by the true stress–true strain relationship used; the parameter ‘c’ is used to adjust the effect of yield on the third invariant of the stress offset tensor; ${\bar{\sigma }}_{D}$ represents the effective stress of Drucker; and the parameters s_1_, s_2_, and s_3_ are the principal values of the deviatoric stress tensor. The linear transformation stress tensor is expressed as follows:

(13) s=L σ

(14) $$L=\left[\begin{array}{cccccc} \frac{\left(c_{2}+c_{3}\right)}{3} & \frac{-c_{3}}{3} & \frac{-c_{2}}{3} & 0 & 0 & 0\\ \frac{-c_{3}}{3} & \frac{\left(c_{3}+c_{1}\right)}{3} & \frac{-c_{1}}{3} & 0 & 0 & 0\\ \frac{-c_{2}}{3} & \frac{-c_{1}}{3} & \frac{\left(c_{1}+c_{2}\right)}{3} & 0 & 0 & 0\\ 0 & 0 & 0 & c_{4} & 0 & 0\\ 0 & 0 & 0 & 0 & c_{5} & 0\\ 0 & 0 & 0 & 0 & 0 & c_{6} \end{array}\right]$$

(15) $$\sigma ={\left(\sigma_{\mathrm{xx}},\;\sigma_{\mathrm{yy}},\;\sigma_{\mathrm{zz}},\sigma_{\mathrm{yz}},\sigma_{\mathrm{zx}},\sigma_{\mathrm{xy}}\right)}^{T}$$

For cases where the yield function in Equation (10) is strictly semi-convex, Cazacu and Balart extended the Drucker yield function to anisotropy in 2001 [12] in the following form:

(16) $$f\left(\sigma_{\mathrm{ij}}\right)=a{\left({J^{\prime }}_{2}^{3}-c\;J\prime_{3}^{2}\right)}^{\frac{1}{6}}$$

There are eight parameters in the extended Drucker yield function described above. The parameter c is set to 1.226 and 2 for BCC and FCC metals, respectively. The other six parameters in *L* are used to describe the anisotropic behavior of metals, and their values need to be determined experimentally.

### 3.3. Yld91 Function

In 1991, Balart et al. proposed a yield function based on a linear transformation [4]. It is capable of exhibiting a small radius of curvature near uniaxial and biaxial tensile stress states. Compared with the yield surface based on crystallographic calculation, the mathematical expression is simple, and the anisotropic parameters are easy to measure. Researchers can better describe the anisotropic characteristics in the sheet metal surface. Its expression is as follows:

(17) $${\left|{S^{\prime }}_{2}-{S^{\prime }}_{3}\right|}^{m}+{\left|{S^{\prime }}_{3}-{S^{\prime }}_{1}\right|}^{m}+{\left|{S^{\prime }}_{1}-{S^{\prime }}_{2}\right|}^{m}=2{\bar{\sigma }}^{m}$$

where ${\bar{\sigma }}^{\;}$ is the effective stress; m is set to be 6 for BCC metals and 8 for FCC metals; and S_1’_, S_2’_, and S_3’_ are the three principal values of the isotropic plastic equivalent (IPE) transformed stress tensor S’, which is computed as follows:

(18) $$S^{\prime }=L^{\prime }\sigma^{\prime }$$

(19) $$L^{\prime }=\left[\begin{array}{cccccc} \frac{\left(C_{2}+C_{3}\right)}{3} & \frac{{-C}_{3}}{3} & \frac{-C_{2}}{3} & 0 & 0 & 0\\ \frac{-C_{3}}{3} & \frac{\left(C_{3}+C_{1}\right)}{3} & \frac{-C_{1}}{3} & 0 & 0 & 0\\ \frac{-C_{2}}{3} & \frac{-C_{1}}{3} & \frac{\left(C_{1}+C_{2}\right)}{3} & 0 & 0 & 0\\ 0 & 0 & 0 & C_{4} & 0 & 0\\ 0 & 0 & 0 & 0 & C_{5} & 0\\ 0 & 0 & 0 & 0 & 0 & C_{6} \end{array}\right]$$

(20) $$\sigma^{\prime }={\left({\sigma^{\prime }}_{xx},{\sigma^{\prime }}_{yy},{\sigma^{\prime }}_{zz},{\sigma^{\prime }}_{yz},{\sigma^{\prime }}_{zx},{\sigma^{\prime }}_{xy}\right)}^{T}$$

## 4. Characterization of Plastic Behavior

### 4.1. Calibration of Different Plastic Works

**Figure 4:**
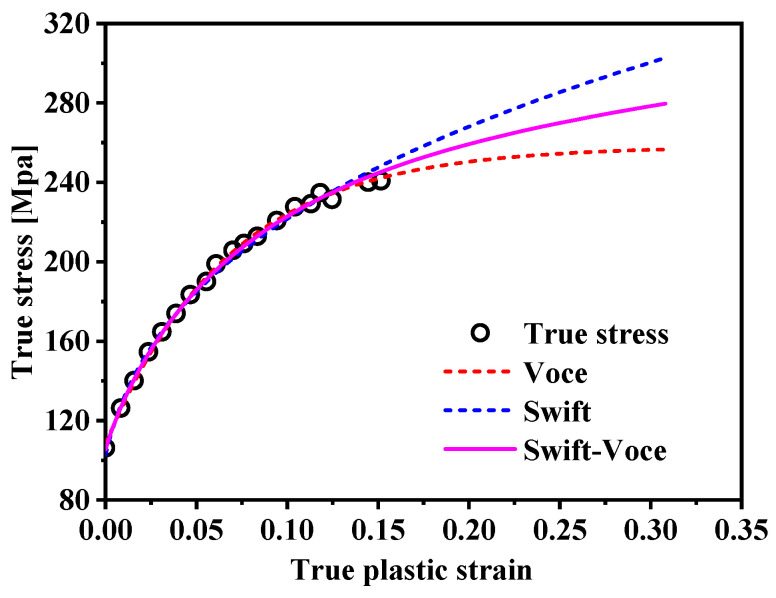
Fitting curves of AA5754-H111 by three hardening criteria.

In metallic materials, the three most widely used classical isotropic hardening models are the Swift criterion in Equation (21), the Voce criterion in Equation (22), and the Swift–Voce hardening criterion in Equation (23), as below:

(21) $$\bar{\sigma }=K{\left(e_{0}+{\bar{\varepsilon }}^{p}\right)}^{n}$$

(22) $$\bar{\sigma }=A-\left(A-B\right)\exp\left(-C{\bar{\varepsilon }}^{p}\right)$$

(23) $$\bar{\sigma }=\alpha [K{\left(e_{0}+{\bar{\varepsilon }}^{p}\right)}^{n}]+\left(1-\alpha \right)[\exp(-C{\bar{\varepsilon }}^{p})]$$

where n is the hardening index; ${\bar{\varepsilon }}^{p}$ is an equivalent plastic strain; K, e_0_, A, B, and C are material constants, and α is a scale factor. The calibration results of the dogbone test using the three hardening models are shown in Figure 4, and we can easily find that the Swift–Voce model fits best. The calibration parameters are presented in Table 3.

**Figure 5:**
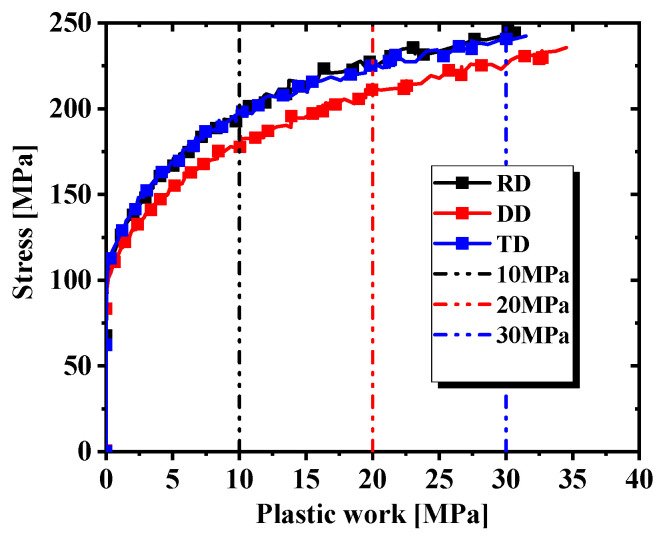
Plastic work evolution in three directions.

**Figure 6:**
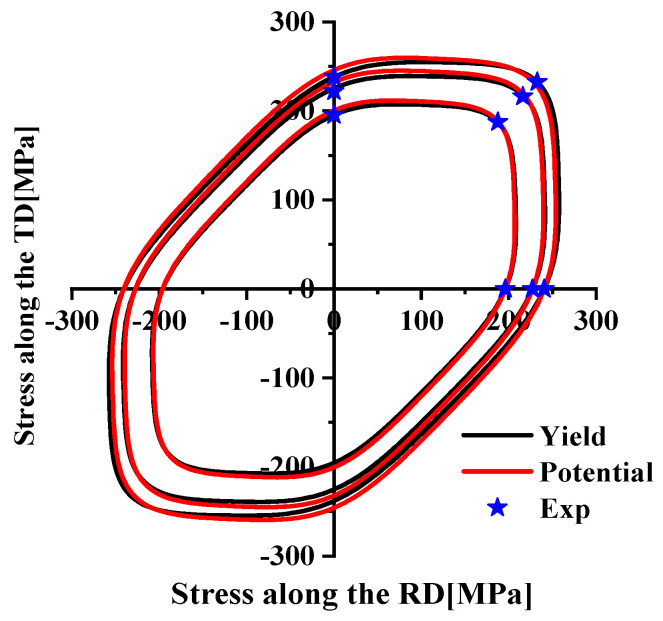
The yield surface calibration of the Drucker model under three plastic works.

The plastic behavior of AA5754-H111 was characterized based on three different plastic works (10 MPa, 20 MPa, 30 MPa), as indicated in Figure 5. The yield stress normalization results of each angle corresponding to the three plastic works are shown in Table 4. The non-AFR was used to perform the plastic calibration of different plastic works for the Drucker yield criterion in Equation (16), where a = 1 and c = 2. The yield surfaces obtained by the calibration of three plastic works are plotted in Figure 6, and the yield parameters are summarized in Table 5. The yield surfaces of the three predictions in Figure 6 all fit perfectly with the test values.

**Figure 7:**
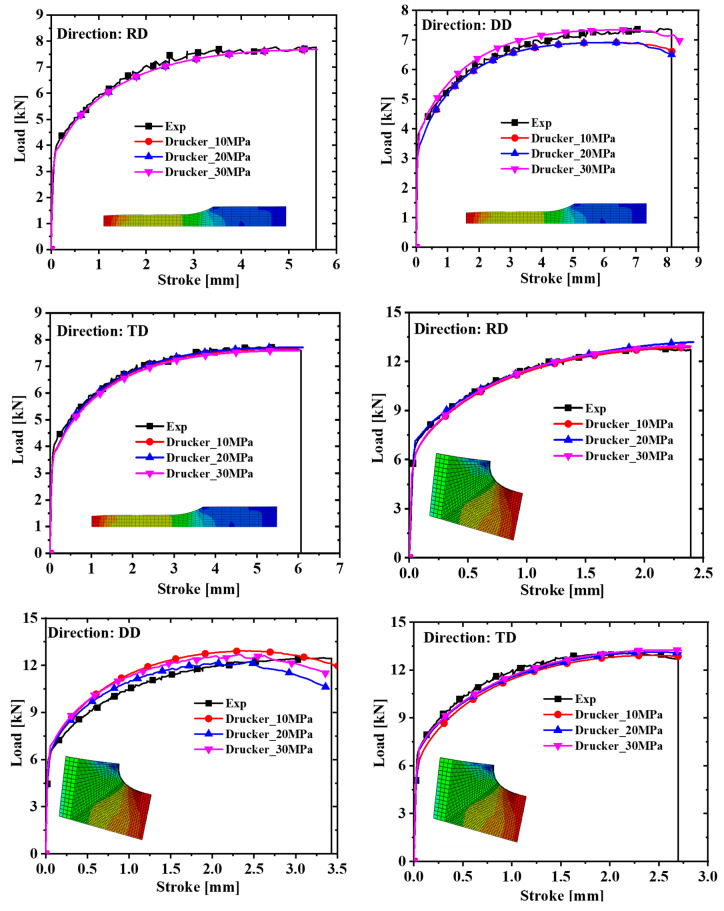
Comparison of force–displacement curves of dogbone specimens and notched specimens with R = 5 mm under different plastic works.

**Figure 8:**
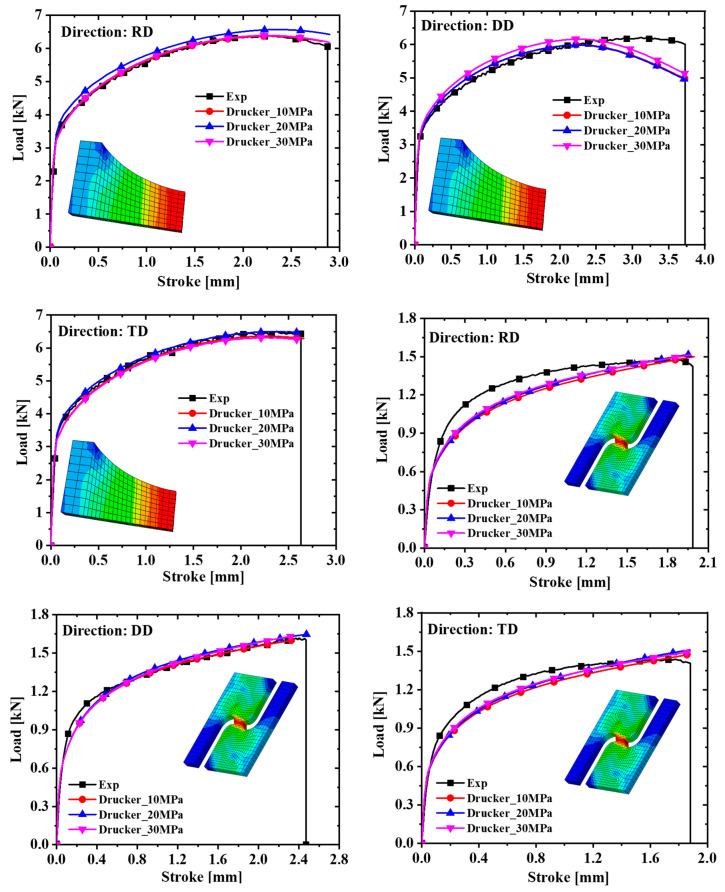
Comparison of force–displacement curves of notched specimens with R = 20 mm and in-plane shear specimens under different plastic works.

Hypermesh software was used to model and mesh the four specimens in three dimensions, and the mesh type of the finite element model was C3D8R. The symmetry of the specimen was fully exploited in order to reduce the calculation time. In the simulation process, the dogbone, R5, and R20 specimens adopted the 1/8 model, and the shear specimens took the 1/2 model. The calibration parameters and the finite element model were imported into the finite element simulation software to complete the tensile test simulation. The Poisson ratio was set to 0.33 and the elastic modulus was 69,000 MPa. The comparison of the force–displacement curves and the test values extracted from the simulation are shown in Figure 7 and Figure 8. It is concluded from Figure 7 and Figure 8 that the prediction of the shear specimen in the 0° direction is not very ideal, but the overall prediction effect is relatively accurate. The prediction result is best when the plastic work is 30 MPa.

### 4.2. Calibration of Different Models

**Figure 9:**
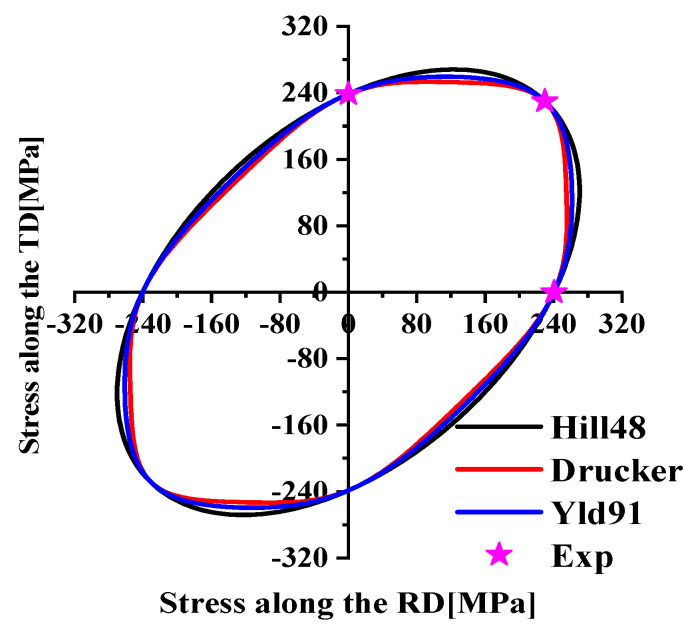
Yield surfaces of the Hill48, Drucker, and Yld91 models.

**Figure 10:**
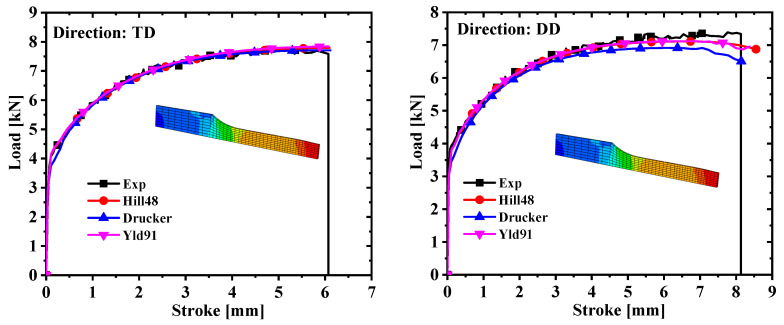

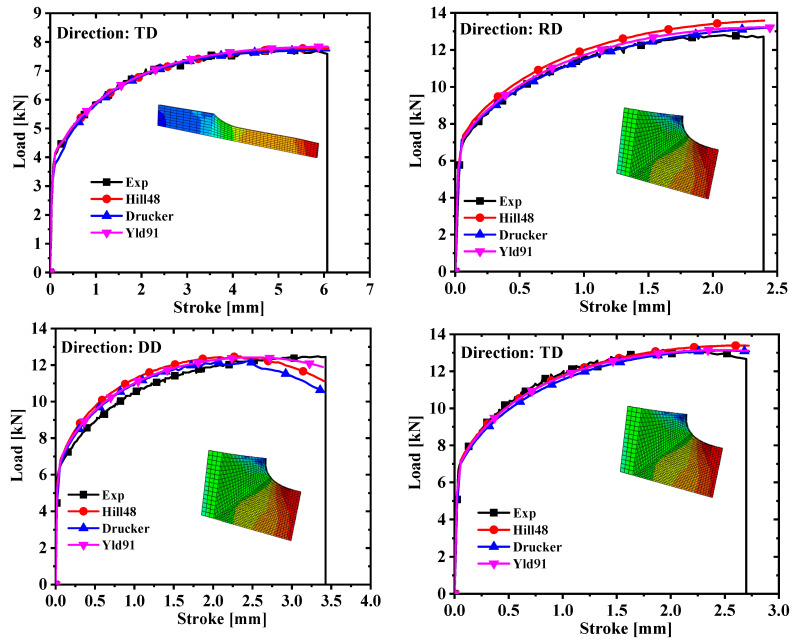
Comparison of force–displacement curves of dogbone specimens and notched specimens with R = 5 mm under different models.

**Figure 11:**
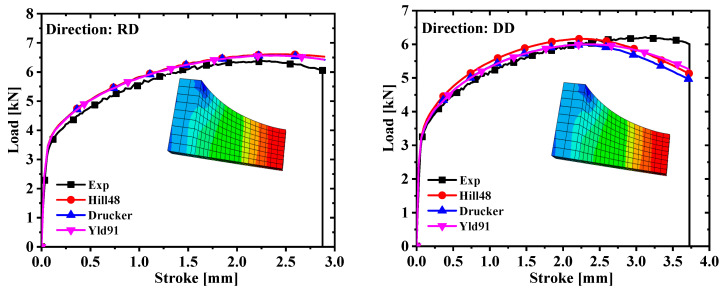

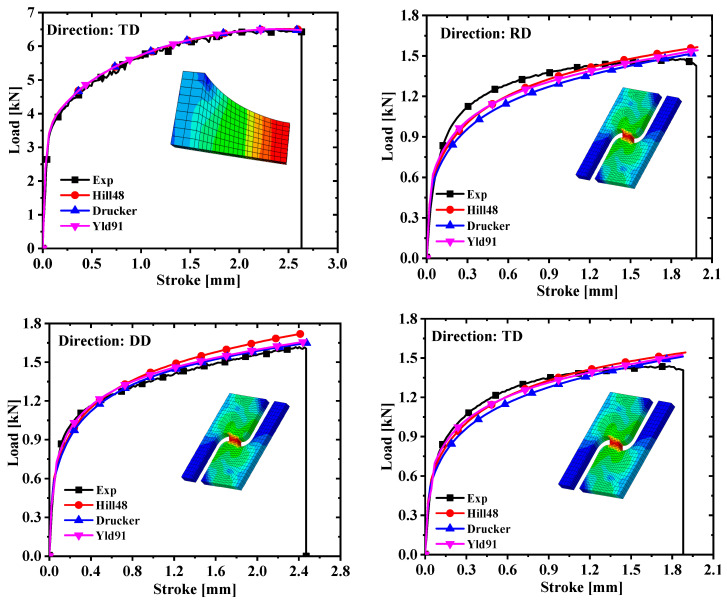
Comparison of force–displacement curves of notched specimens with R = 20 mm and in-plane shear specimens under different models.

The yield stress with a plastic work of 30 MPa was selected based on the above conclusions, and the anisotropic yield parameters of the Hill48 and Yld91 yield criteria were further calibrated. To facilitate the comparison of the accuracy of the three yield criteria, calibration was also performed under the non-AFR. Since AA5754-H111 is an FCC metal, m = 8 in the Yld91 yield criterion. The yield surface and test values calibrated by the yield criteria of Hill48, Drucker, and Yld91 are indicated in Figure 9. It is seen from Figure 9 that the yield surface of the three yield criteria does not match the test values well, and the yield surface of the Hill48 model is slightly larger. The anisotropic parameters of the Hill48 and Yld91 models are shown in Table 6. Comparing the simulated values with the experimental values in Figure 10 and Figure 11, it is observed that the prediction results of the Hill48 model are slightly higher than those of the other two models. In general, the Yld91 model and the Drucker model have high prediction accuracy.

## 5. Cylinder Drawing Verification

### Cylinder Drawing Test

**Figure 12:**
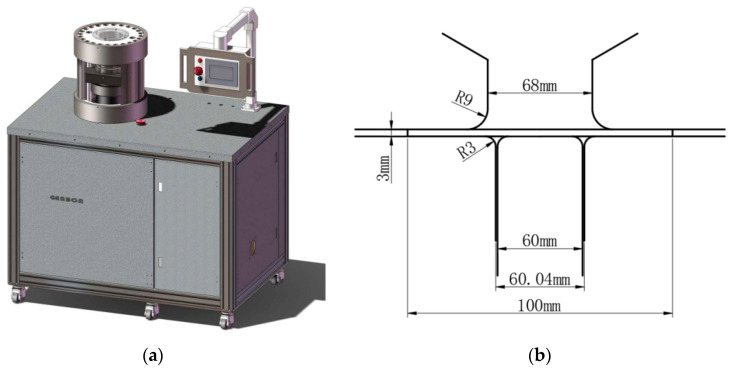
Forming testing machine and mold size drawing: (**a**) forming testing machine; (**b**) dimensional drawing of the cylinder drawing fixture.

In order to verify the accuracy of the above three models for calibration of the anisotropic yield of AA5754-H111, the cylinder drawing test was studied. The dimensional schematic diagrams of the forming limit testing machine and the test device are presented in Figure 12. The diameter of the punch was 60 mm, and the diameter of the die was 68 mm. The laser cutting method was used on the plate to process a cylindrical specimen with a diameter of 100 mm. The rolling direction was marked on the cylindrical specimen before the test so that the height of the earing in different directions could be measured after the test. The influence of lubrication work on the cylinder drawing test is crucial, and petroleum jelly was evenly applied to the upper and lower surfaces of the center of the specimen and the surface of the hammer to reduce friction before the experiment. Since the position of the cylindrical specimen on the mold has a serious impact on the test results, the position of the specimen was calibrated using a three-jaw chuck to ensure that the specimen was in the center of the mold. A constant edge pressing force of 10 kN was maintained throughout the test to prevent warpage around the specimens. The cylindrical punch hammer moved down at a constant speed of 1 mm/s.

**Figure 13:**
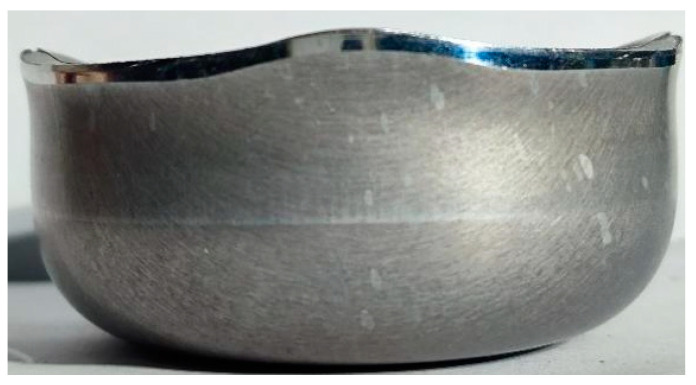
Cylindrical specimen after testing.

**Figure 14:**
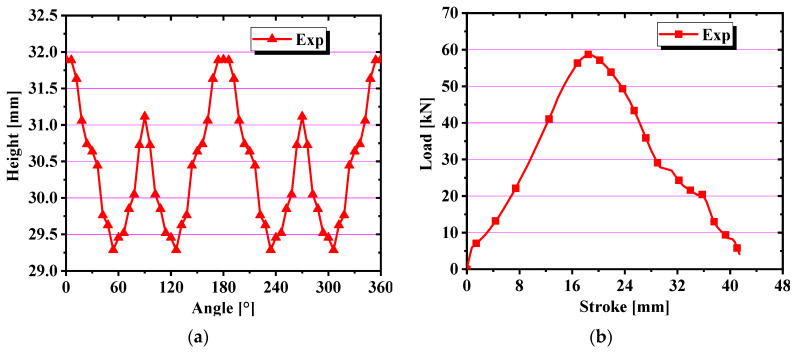
(**a**) Earing height evolution with angle; (**b**) force–displacement curve for cylinder deep drawing test.

**Figure 15:**
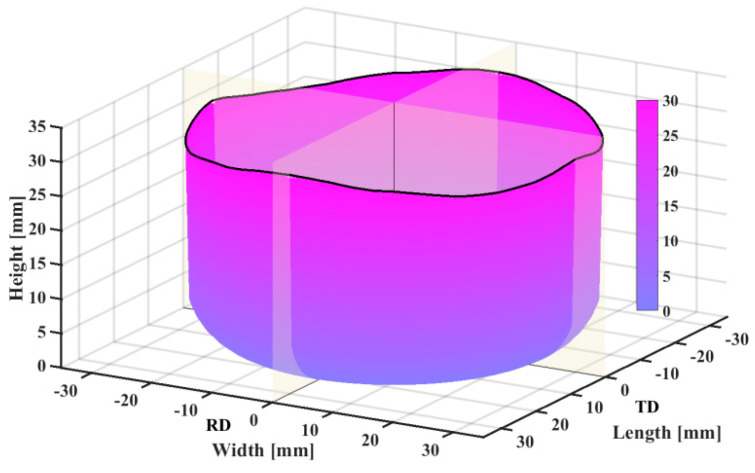
Force–displacement curve for cylinder deep drawing test.

There is an obvious earing phenomenon, and the four ears are symmetrically distributed in Figure 13. The experiment was conducted at room temperature and the punch velocity was very slow at 1.0 mm/s. Therefore, there was approximately no temperature change during the tests. The variation trend and force–displacement curve of earing height with increasing angle are shown in Figure 14. A three-dimensional diagram was drawn from the experimental data, as shown in Figure 15, which is convenient for us to observe the position and height of the earing distribution. It is obvious that earing peaks appear at 0°, 90°, 180°, and 270° and earing valleys at angles of 45°, 135°, 225°, and 315° from Figure 14 and Figure 15.

**Figure 16:**
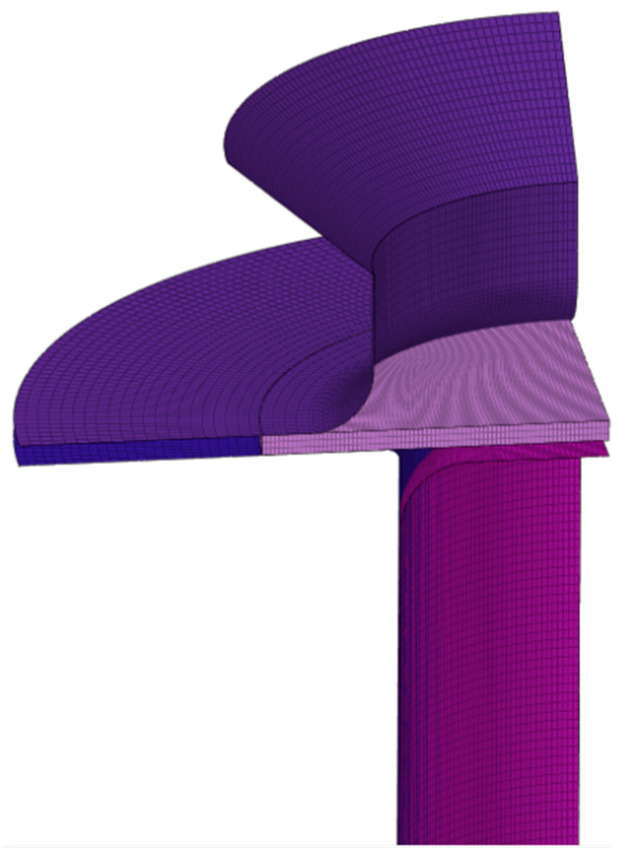
Cylinder drawing finite element model.

This part of the simulation used the AFR and the non-AFR to compare the cylinder drawing simulation under different yield models and flow rules. The AA5754-H111 cylinder drawing simulation was performed on the commercial finite element platform LSDYNA/Explicit. The three anisotropic yield models of Hill48, Drucker, and Yld91 were implemented by LSDYNA’s own material cards, which are material cards 122, 263, and 33. The dimensions of the finite element model are illustrated in Figure 12. In order to reduce the finite element simulation time, a quarter finite element model was established, as plotted in Figure 16. The pressing force in the simulation was also a quarter of 2.5 kN at the test and was applied to the crimping ring. Only the test piece was defined as deformable, and all other parts were defined as rigid meshes for simulation. The mesh type was a three-dimensional hexahedral solid element (C3D8R). The coefficient of friction between the punch and the specimen was defined as 0.2, and the Coulomb coefficient of friction for other contact pairs was defined as 0.1. The maximum displacement of the test piece in the test was 41.5 mm. The displacement of the punching hammer in the simulation must be greater than the test value to ensure the success of the simulation. Taking into account the initial spacing between the hammer and the test piece, the displacement of the hammer in the simulation was set to 60.0 mm.

**Figure 17:**
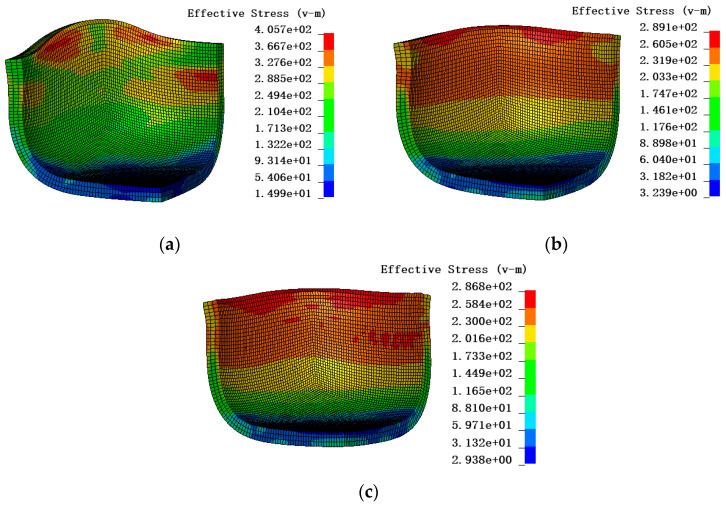
Cylinder drawing simulation of different models under the AFR: (**a**) Hill48; (**b**) Drucker; (**c**) Yld91.

**Figure 18:**
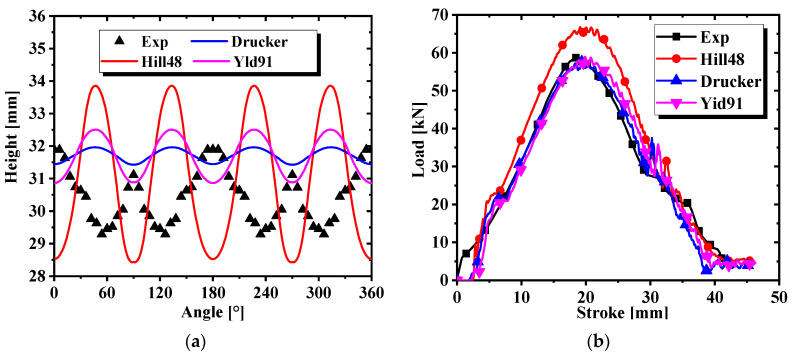
Predictions of three models under the AFR: (**a**) change in earing height with angle change; (**b**) force–displacement curves for cylinder drawing simulation.

**Figure 19:**
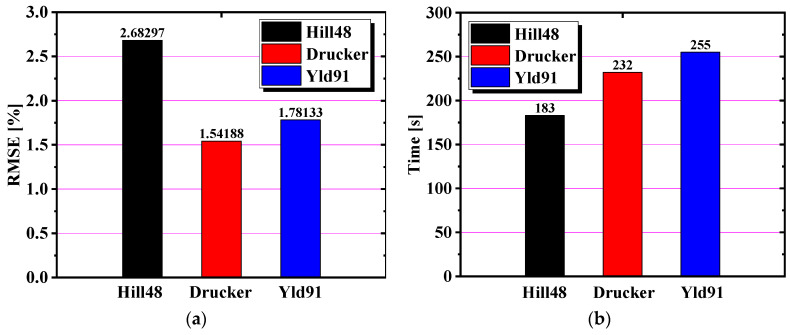
Comparison of accuracy and time of the three models under the AFR: (**a**) the RMSE of the cylinder drawing simulation; (**b**) the time of the cylinder drawing simulation.

The anisotropic yield parameters of the three models of yield stress calibration in Table 5 and Table 6 were entered into the material cards No. 122, No. 263, and No. 33 under the AFR. However, the anisotropic yield parameters of yield stress calibration were not applied to material card No. 122, and the anisotropic yield parameters of yield stress calibration needed to be converted into R-values by Equations (7)–(9). The stress cloud diagrams of the finite element simulation are shown in Figure 17. The maximum effective stress was about 405.7 MPa for Hill48, 289.1 MPa for Drucker, and 286.8 MPa for Yld91. The effective stress predicted by the Hill48 model is much greater than that of the other two models. The height and force–displacement curves of the earing at different angles were extracted in the simulation results, as indicated in Figure 18. It is clear from Figure 18a that the angle-based prediction of the appearance of earing of the three materials stuck to the cylinder drawing test under the AFR is not accurate. In Figure 18b, it is found that material card No. 263 and material card No. 33 are more accurate in predicting the force–displacement curve of the test, but material card No. 122 does not predict the force–displacement curve of the test accurately. To make the simulation and test errors more obvious, the root mean square errors of the earing prediction values of the above three material cards were calculated, as detailed in Figure 19a. In addition, the simulation time of the three material cards is compared in Figure 19b, where material card No. 122 shows the shortest time, and material cards No. 33 and No. 263 show close times.

**Figure 20:**
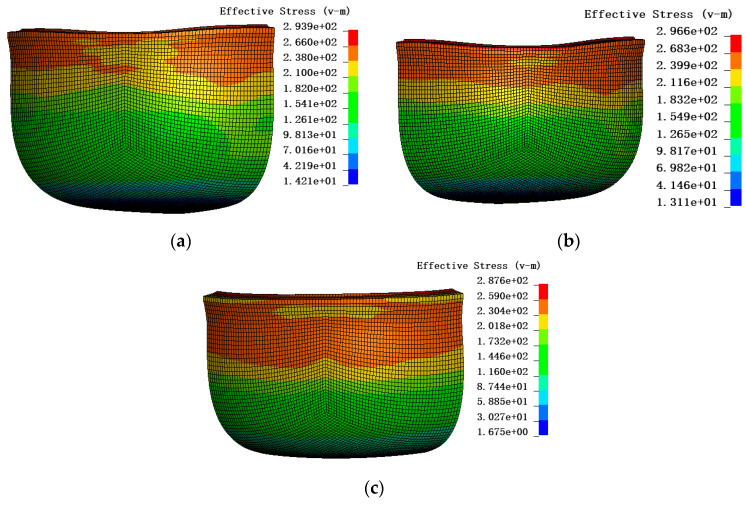
Stress clouds for three cases: (**a**) AYAPP; (**b**) AYIPP; (**c**) IYAPP.

In order to facilitate the comparison of the AFR and the non-AFR, the earing behavior of the cylinder deep drawing test was studied under the non-AFR conditions. In the LSDYNA/Explicit finite element simulation software, the non-AFR cannot be used for material cards 122 and 33. Therefore, this part of the simulation mainly revolved around material card No. 263. Based on material card No. 263, the effects of the yield function and the plastic potential function on earing behavior were compared. The anisotropic yield function and the anisotropic plastic potential function (AYAPP), the anisotropic yield function and the isotropic plastic potential function (AYIPP), and the isotropic yield function and the anisotropic plastic potential function (IYAPP) were used for the simulations. The parameters of the above three cases are calibrated in Table 7. These parameters were entered in material card No. 263 to obtain the simulated cloud diagram shown in Figure 20. It is found that the difference in effective stress in the three cases is small in Figure 20.

**Figure 21:**
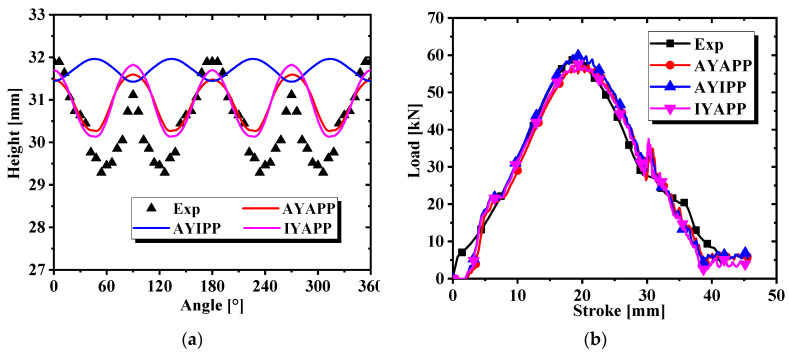
Predictions of three cases under the non-AFR: (**a**) change in earing height with angle change; (**b**) force–displacement curves for cylinder drawing simulation.

**Figure 22:**
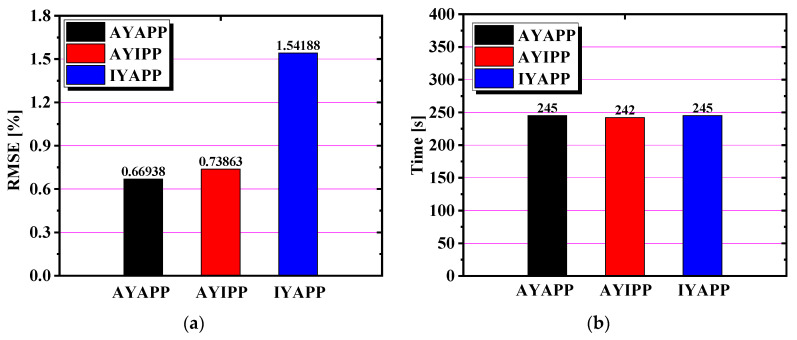
Comparison of accuracy and time of the three situations under the non-AFR: (**a**) the RMSE of the cylinder drawing simulation; (**b**) the time of the cylinder drawing simulation.

The earing height prediction values and the simulated force–displacement curve of the earing can be obtained from the simulation results in Figure 21. It is observed from Figure 21a that AYIPP cannot predict the position of the earing, IYAPP predicts the position of the earing very well but does not accurately predict the height of the earing, and AYAPP predicts the position and height of the earing accurately. The force–displacement curves predicted by the three conditions match with the test curves well in Figure 21b. The prediction curves of IYAPP and AYAPP are relatively close, indicating that the plastic potential function is more critical for predicting the formation of earing. The root mean square error plot and simulation time for the above three cases are calculated in Figure 22. It shows that AYAPP has the smallest root mean square error and is closest to the experimental value. The time taken in all three cases is about the same, so the simulation time of the same model under the non-AFR has little to do with the yield model and the plastic potential function.

## 6. Conclusions

This research evaluated the performance of the Hill48, Yld91, and anisotropic Drucker yield functions and different combinations of yield functions and potential functions. Experiments were conducted for four different types of specimens along different directions to characterize the anisotropy and fracture behavior of the alloys. Cup deep drawing tests were conducted and simulated with different constitutive models, and the numerical simulation results were compared with the experimental results to evaluate the performance of the different constitutive models. Based on the comparison, the following conclusions are obtained:

1. In the tensile test of AA5754-H111, it was found that the alloy has strong anisotropy under different stress conditions. Based on the non-AFR criterion, the Drucker yield model was applied to three plastic works, and the prediction was most accurate when the plastic work was equal to 30 MPa. The Hill48, Drucker, and Yld91 models were applied to AA5754-H111, and the results showed that the accuracy of the Hill48 model is slightly inferior to that of the Drucker and Yld91 yield models.
2. On the basis of the AFR, LSDYNA’s material cards were used for cylinder drawing simulations. The study found that Hill48 has the shortest simulation time and anisotropic Drucker and Yld91 models take longer. The predictions for the force–displacement curve were the opposite, with the former having the worst accuracy and the latter two having higher accuracy.
3. The presence of four symmetrically distributed lugs in the cylinder drawing test of AA5754-H111 verified the rationality of the three yield models. In the cylinder drawing finite element simulation, it was observed that the AFR does not accurately predict the location of the earing appearance, while the non-AFR predicts it accurately. The comparison highlights the superiority of the non-AFR.
4. Based on material card No. 263 in LSDYNA/Explicit finite element software, it was found that IYAPP is closer to AYAPP in the simulation results. It is thus concluded that the plastic potential function has a greater influence on the prediction of earing behavior in the cylinder deep drawing test than the yield function.

## Figures and Tables

**Table 1:** Four types of specimens’ tensile speed [mm/min].

Specimens	Dogbone	R5	R20	Shear
Velocity	3.6	0.5	0.5	0.5

**Table 2:** The R-values of the dogbone specimens in three directions.

	RD	DD	TD
Test1	0.61309	0.24905	0.77390
Test2	0.64696	0.26373	0.70593
Test3	0.67062	0.26352	0.70346
Average R-value	0.64356	0.25877	0.72776

**Table 3:** Hardening parameters for the three hardening criteria.

Hardening Criteria	K[GPa]	e_0_	n	A[GPa]	B[GPa]	C
Swift–Voce	0.41066	0.00585	0.27479	0.25912	0.10863	14.5622

**Table 4:** Yield stress corresponding to three plastic works.

Plastic Work [MPa]	RD [MPa]	DD [MPa]	TD [MPa]
10	1.0000	0.91438	0.99674
20	1.0000	0.91404	0.99168
30	1.0000	0.93776	0.98994

**Table 5:** The yield parameters of the Drucker model under three plastic works.

Plastic Work [MPa]	Function	c_1_	c_2_	c_3_	c_4_	c_5_	c_6_
10	Potential	1.87994	1.95489	1.71211	1.53126	1.53126	1.53126
	Yield	1.92271	1.91779	1.75244	2.03856	2.03856	2.03856
20	Potential	1.87994	1.95489	1.71211	1.53126	1.53126	1.53126
	Yield	1.93807	1.90820	1.76267	2.03612	2.03612	2.03612
30	Potential	1.87994	1.95489	1.71211	1.53126	1.53126	1.53126
	Yield	1.91863	1.88210	1.79007	1.97689	1.97689	1.97689

**Table 6:** Yield parameters for Hill48 and Yld91 models.

Model	Function	**F**	**G**	**H**	**L**	**M**	**N**
Hill48	Potential	0.53814	0.60843	0.39156	0.86478	0.86478	0.86478
	Yield	1.13400	1.07970	0.92030	3.69000	3.69000	3.69000
	Function	***C*_1_**	***C*_2_**	***C*_3_**	***C*_4_**	***C*_5_**	***C*_6_**
Yld91	Potential	1.02781	1.07896	0.91742	0.80470	0.80470	0.80470
	Yield	1.05537	1.03897	0.96017	1.10894	1.10894	1.10894

**Table 7:** Yield parameters for AYAPP, AYIPP, and IYAPP.

	Function	c_1_	c_2_	c_3_	c_4_	c_5_	c_6_
AYAPP	Yield	1.91863	1.88210	1.79007	1.97689	1.97689	1.97689
	Potential	1.87994	1.95489	1.71211	1.53126	1.53126	1.53126
AYIPP	Yield	1.91863	1.88210	1.79007	1.97689	1.97689	1.97689
	Potential	1.86350	1.86350	1.86350	1.86350	1.86350	1.86350
IYAPP	Yield	1.86350	1.86350	1.86350	1.86350	1.86350	1.86350
	Potential	1.87994	1.95489	1.71211	1.53126	1.53126	1.53126

## Data Availability

Data is unavailable due to privacy.
